# Accurately Identifying Risk Factors Specific to Women is Crucial to Reducing Cardiovascular Disease Risk

**DOI:** 10.1097/jnr.0000000000000699

**Published:** 2025-08-01

**Authors:** Chii JENG

Cardiovascular disease (CVD) is the leading cause of death globally, responsible for nearly 18 million deaths annually. The World Health Organization predicts that the global burden of CVD will increase substantially by 2050. Specifically, the prevalence of CVD is projected to increase by 90.0%, and the crude mortality rate is projected to increase by 73.4% (Chong et al., 2024). Individuals with CVD are at high risk of major adverse cardiac events (MACEs) such as myocardial infarction, stroke, heart failure, and cardiac death. Even 4–5 years after a myocardial infarction, the risk of experiencing a major adverse cardiac event remains elevated. Furthermore, CVD is associated with the highest economic burden among diseases globally, surpassing Alzheimer’s disease and diabetes.

In terms of prevalence, women and men are nearly equally affected by CVD. World Health Organization data reveal that 43% of those who died from ischemic heart disease in 2020 were women, making CVD the leading cause of death among women globally. In 2021, the Lancet Commission reported that, despite it being the primary cause of death among women globally, CVD remains underdiagnosed, undertreated, and under-researched. This lack of focus on CVD in women has resulted in a global deficiency in reliable data on disease incidence and outcomes, insufficient research and awareness, inadequate diagnostic and therapeutic approaches, and limited understanding of the sex-specific mechanisms of CVD pathophysiology and natural history. Furthermore, the incidence of myocardial infarction and CVD mortality has been rising among younger women, while female representation in cardiovascular clinical trials remains low. Therefore, improving cardiovascular health among women is essential to reducing the global burden of CVD by 2030 (Vogel et al., 2021).

Cardiovascular risk factors differ by gender, and several risk factors, including gestational hypertension, gestational diabetes, preeclampsia, and premature menopause, are specific to women (Wenger et al., 2022). Nevertheless, widely used cardiovascular risk assessment tools such as the Framingham Risk Score developed from the Framingham Heart Study and the Atherosclerotic Cardiovascular Disease Risk Score Calculator jointly established by the American College of Cardiology and the American Heart Association do not consider female-specific risk factors. Currently, no dedicated risk assessment tool is available that specifically addresses the cardiovascular risk factors unique to women. In the current era of precision medicine, establishing a cardiovascular risk scoring system tailored to women is a pressing concern that must be addressed. Therefore, developing and applying a precise assessment system that considers sex-specific risk factors will be critical to effectively reducing the global burden of CVD.
